# Endometrial Carcinoma in a 26-Year-Old Patient with Bardet-Biedl Syndrome

**DOI:** 10.1155/2018/1952351

**Published:** 2018-05-03

**Authors:** Olga Grechukhina, Gregory M. Gressel, William Munday, Serena Wong, Alessandro Santin, Alla Vash-Margita

**Affiliations:** ^1^Department of Obstetrics, Gynecology and Reproductive Sciences, Yale University School of Medicine, 333 Cedar Street, FMB307, New Haven, CT 06520, USA; ^2^Montefiore Medical Center, Division of Gynecologic Oncology, Department of Obstetrics & Gynecology and Women's Health, 1300 Morris Park Avenue, Belfer 501, Bronx, NY 10461, USA; ^3^Department of Pathology, Yale University School of Medicine, 310 Cedar Street, LH 108, New Haven, CT 06520, USA

## Abstract

**Background:**

Bardet-Biedl Syndrome (BBS) is a rare genetic condition characterized by cognitive impairment, dysmorphism, central obesity, and diabetes mellitus, among other abnormalities. Although some of these characteristics are known independent risk factors for endometrial cancer and its precursors, the association between BBS and endometrial cancer is underreported.

**Case:**

We present the case of a 26-year-old patient with BBS and clinical signs of hyperestrogenism who presented with abnormal uterine bleeding and was diagnosed with endometrioid adenocarcinoma. She ultimately underwent definitive surgical treatment with hysterectomy and bilateral salpingectomy.

**Conclusions:**

This is one of only a few reports in the literature describing the association of BBS and endometrioid endometrial adenocarcinoma. Given the association of BBS with risk factors for hyperestrogenism such as truncal obesity, hyperinsulinemia, and ovulatory dysfunction, providers should have increased suspicion for endometrial cancer in young patients with BBS and abnormal uterine bleeding.

## 1. Introduction

Bardet-Biedl syndrome (BBS), first described in 1920, is a rare autosomal recessive disorder, the prevalence of which ranges from 1 : 140,000 to 160,000 in North American and European populations but is much more common in certain Arab groups [1]. It is characterized by a spectrum of clinical features including progressive retinal degeneration, central obesity, limb abnormalities, cognitive impairment, and gonadal and renal dysgenesis as well as speech problems, developmental delay, diabetes mellitus, diabetes insipidus, heart disease, and many others. Here we present a case report of a woman with BBS who presented for evaluation of abnormal uterine bleeding and ultimately was diagnosed with uterine cancer at the age of 26.

## 2. Case Report

We present a 26-year-old Hispanic gravida 0 with a known history of Bardet-Biedl syndrome and abnormal uterine bleeding (AUB). She was evaluated in the emergency room (ER) for heavy vaginal bleeding and dizziness and was found to have profound anemia with a hemoglobin and hematocrit of 5.4 g/dL and 15%, respectively. The patient was seen in the ER one week prior with same symptoms and was then started on conjugated equine estrogen (Premarin 5 mg twice daily). Her gynecologic history was notable for menarche at the age of 13 followed by regular periods until 2 years prior to her presentation. She remained amenorrheic for 1.5 years until she resumed regular 5-day periods with normal flow 6 months ago. The patient had been sexually active but denied history of sexually transmitted diseases. Review of her records revealed three prior abnormal Pap smears (abnormal squamous cells of undetermined significance in association with positive high-risk HPV testing). Three prior colposcopies with cervical biopsies were negative.

The patient's past medical history included Bardet-Biedl syndrome, morbid obesity with body mass index (BMI) of 54 kg/m^2^, severe persistent asthma, congenital absence of one kidney, obstructive sleep apnea, and scoliosis. She reported no pertinent past surgical or social history. Her family history was notable for unprovoked deep vein thrombosis in the patient's sister. She denied any known medical allergies. Her medication list included conjugated estrogen (Premarin) 5 mg twice daily, fluticasone/salmeterol (Advair) twice daily, and albuterol (Proventil) as needed. Social history was unremarkable.

**Figure 1:**
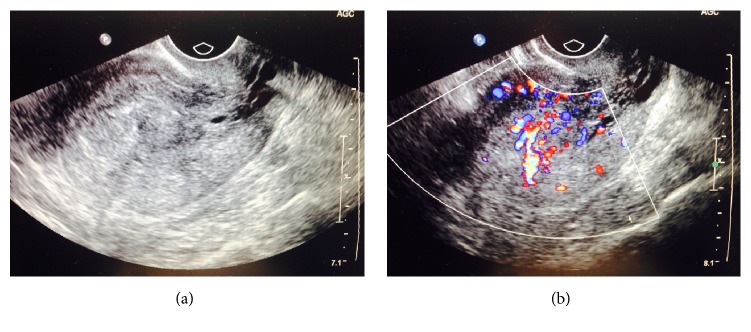
Transvaginal ultrasound examination of pelvic organs. (a) Sagittal view of the uterus with thick mildly heterogeneous endometrium measuring 1.6 cm, without discrete polyp or mass lesion. (b) Possible hypervascular stalk on color Doppler evaluation suggestive of possible endometrial mass.

On presentation to the ER, the patient's vital signs included blood pressure of 132/66 mmHg, heart rate of 115 bpm, respiratory rate of 16, oxygen saturation of 98%, temperature of 37.6 C, and weight of 146.5 kg. Her physical exam was notable for morbid obesity, no abdominal tenderness, and a 30 mL blood clot at cervical os. Bimanual exam demonstrated closed nontender cervix. Fundus and adnexa were not palpated due to body habitus. Laboratory findings were otherwise unremarkable except for anemia indices mentioned above. Urine pregnancy test was negative. Pelvic anatomy was evaluated with transvaginal sonography, which revealed a thickened, 1.6 cm mildly heterogeneous and hypervascular endometrium without identification of a discrete mass but with a possible hypervascular stalk suggestive of polyp (Figure 1). The uterus measured 8.3 × 5.1 × 5.3 cm and was mildly heterogeneous. Right ovary appeared morphologically normal, whereas the left ovary was not visualized. She was admitted to gynecology service for further management. Patient was transfused 2 units of packed red blood cells. Medroxyprogesterone (Depo-Provera) 150 mg IM was given to stabilize her endometrium, and patient was started on medroxyprogesterone 20 mg orally every 8 hours with quick control of her bleeding.

Patient subsequently underwent hysteroscopy, dilation, and curettage which revealed hypertrophic, vascular appearing endometrial tissue with presumed endometrial polyp. Patient was then discharged home on oral medroxyprogesterone and close follow-up.

**Figure 2:**
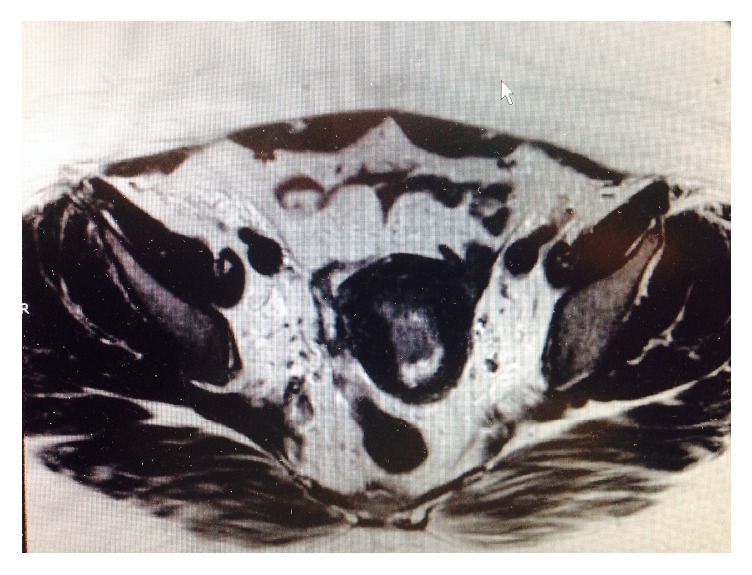
Magnetic resonance imaging of pelvic organs with Gadavist intravenous contrast demonstrated a 3.5 × 1.2 × 1.5 cm T2 enhancing mass with less than 50% invasion into the myometrium. No evidence of local regional metastatic disease.

**Figure 3:**
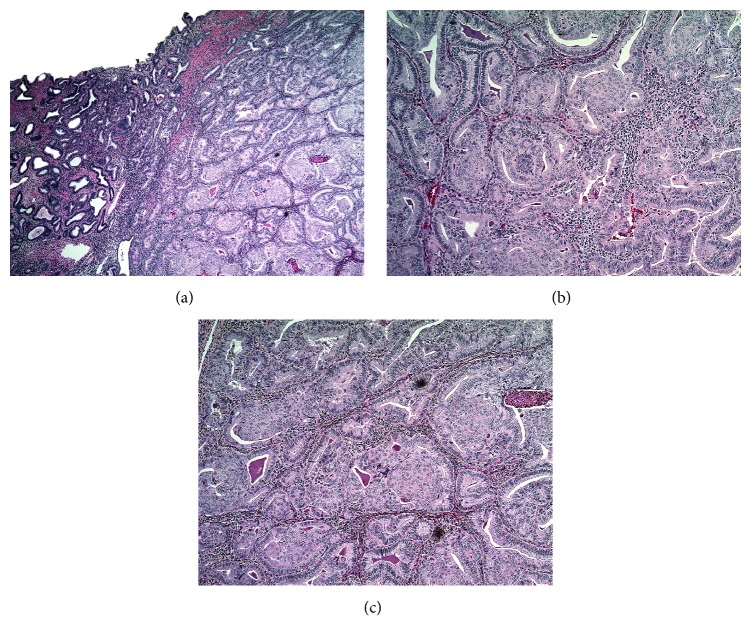
Endometrial adenocarcinoma, endometrioid type with squamous differentiation. (a) Sharp demarcation between adenocarcinoma and adjacent endometrium showing atypical complex hyperplasia. (b) Squamous metaplasia within endometrioid type adenocarcinoma. (c) Photomicrograph demonstrating endometrioid adenocarcinoma with prominent areas of squamous metaplasia (arrows).

Final pathology from her endometrial sampling was reported as FIGO grade 1, nuclear grade 1 endometrioid adenocarcinoma with squamous metaplasia. She was then referred to a gynecologic oncologist for further care. Pelvic and abdominal MRI were performed and revealed less than 50% invasion into the myometrium and no apparent extra uterine disease (Figure 2). After extensive discussion the decision was made to proceed with definitive surgical management and the patient underwent robotic assisted total laparoscopic hysterectomy and bilateral salpingectomy with ovarian preservation and bilateral pelvic lymph node dissection with sentinel node biopsy. Intraoperative findings were notable for a 12-week sized globular uterus, normal fallopian tubes and ovaries, and fatty liver. No extra uterine masses were identified. Patient was discharged home on postoperative day 1. Final pathology assessment was FIGO grade 2, nuclear grade 2, endometrioid adenocarcinoma with squamous differentiation, with her background endometrium demonstrating complex atypical hyperplasia. Final TNM staging was pT1aN0, FIGO stage IA (Figure 3). The patient is currently recovering well from the surgery. No further chemotherapy was recommended.

## 3. Discussion

Bardet-Biedl syndrome is a pleotropic genetic disorder, which may present as a wide spectrum of clinical signs. Visual disorders, limb defects, obesity, learning difficulties, renal tract abnormalities, and genital anomalies comprise major criteria. Minor features include developmental delay, neurological and motor deficits, difficult behavior, speech deficits, hearing abnormalities, asthma, abnormal facial features, hypogonadism, diabetes mellitus, and heart defects. Four major features are required for the diagnosis. Early diagnosis remains difficult, as not all of the main features are present at birth. Genital abnormalities are more frequently found in males with BBS. In females reproductive system anomalies may include hypoplastic fallopian tubes, uterus and ovaries, partial or complete vaginal atresia, absent urethral orifice, persistent urogenital sinus, and hydrometrocolpos. Many patients will have irregular periods. Interestingly, female infertility is not necessarily one of the defining features of the syndrome. There are reports of patients with BBS giving birth to healthy offspring [2].

BBS is genetically heterogeneous with associated pathogenic variants in eighteen genes. The pathogenesis of BBS is believed to be related to cilia and intraflagellar transport dysfunction in a similar manner as Kartagener syndrome, autosomal dominant polycystic kidney disease, and nephronophthisis [3]. All known BBS proteins have been found to play an important role in ciliated cells with their function most commonly localized to the basal bodies of the primary cilium. Mutations in BBS genes and thus abnormal BBS proteins result in defects in development and function of such ciliated structures as retina, olfactory nerve, sperm, ventricular lining of the brain, hypothalamic nerves, and others resulting in a typical symptomatic pattern [4].

Obesity in patients with BBS is thought to be related to endogenous leptin resistance with associated hyperleptinemia due to abnormal leptin receptor function in hypothalamus. Interestingly, in hypothalamus many of the leptin responsive neurons are ciliated and BBS proteins are crucial for cilia function [5]. Moreover, it has been shown that cilia is an indispensable part of preadipocyte to adipocyte differentiation and BBS genes are important regulators of this process [6].

It has previously been shown that BMI is strongly associated with type I endometrial cancer. A recent meta-analysis by Jenabi and Poorolajal quotes the estimated relative risk (RR) and odds ratio (OR) of endometrial cancer was 1.34 (95% CI: 1.20, 1.48) and 1.43 (95% CI: 1.30, 1.56) for the overweight and 2.54 (95% CI: 2.27, 2.81) and 3.33 (95% CI: 2.87, 3.79) for the obese, respectively [7]. The mechanism of carcinogenesis in obese women is thought to be related to higher levels of circulating estrogen in postmenopausal women and lower progesterone levels (unopposed estrogen) in premenopausal women. Increased estrogen concentrations result from excessive peripheral conversion of androgens to estrogen in adipose tissue by aromatase. Obesity associated hyperinsulinemia is also known to exert direct growth factor like action on endometrium as well as increasing the levels of bioactive estrogen by decreasing the steroid hormone binding globulin synthesis in the liver. Estrogen stimulates cell proliferation via classical nuclear estrogen receptors (ER) alfa and beta, acting as transcription factors and resulting in increased DNA replication and thus higher risk of genomic mutations. Some studies suggest that a shift in the ratio between two ER subtypes or abnormal functioning of one of them due to epigenetic downregulation in the setting of absolute or relative hyperestrogenism is associated with endometrial carcinogenesis [8].

This is a rare case of a patient with BBS with a diagnosis of endometrial cancer in a 26-year-old patient. To our knowledge, there are only 2 other reports of endometrial cancer in BBS patients, which were described by Peiker et al. and Schwab and Kriz in 1978 and 1980, respectively [9, 10]. Schwab and Kriz described two cases of endometrial cancer diagnosed in two sisters with BBS at the age of 40. The authors of both articles raise question of predisposition of patients with BBS to endometrial cancer. This association may be underreported; thus, the true frequency of this association may be higher. It is unclear if obesity and resulting hyperestrogenism by itself has been a major risk factor in this patient or if this syndrome may play a separate role predisposing for endometrial cancer. This question needs to be studied further. The authors of this manuscript suggest that because women with BBS suffer from other medical conditions that predispose them to hyperestrogenism and endometrial hyperplasia, providers should have increased suspicion for endometrial cancer in women with BBS and abnormal uterine bleeding. There should be low threshold to do endometrial sampling in these patients.

## Additional Points

*Highlights*. (i) Women with Bardet-Biedl syndrome may be at higher risk for endometrial cancer even at young age. (ii) Gynecologic providers should have low threshold to perform endometrial sampling in young women with Bardet-Biedl syndrome who present with abnormal uterine bleeding.
